# The Kappa Opioid Receptor and the Sleep of Reason: Cortico-Subcortical Imbalance Following Salvinorin-A

**DOI:** 10.1093/ijnp/pyab063

**Published:** 2021-09-19

**Authors:** Genís Ona, Frederic Sampedro, Sergio Romero, Marta Valle, Valle Camacho, Carolina Migliorelli, Miguel Ángel Mañanas, Rosa Maria Antonijoan, Montserrat Puntes, Jimena Coimbra, Maria Rosa Ballester, Maite Garrido, Jordi Riba

**Affiliations:** 1 Human Neuropsychopharmacology Group, Sant Pau Institute of Biomedical Research (IIB-Sant Pau), Barcelona, Spain; 2 Sant Pau Institute of Biomedical Research (IIB-Sant Pau), Barcelona, Spain; 3 Centro de Investigación en Red-Enfermedades Neurodegenerativas (CIBERNED), Barcelona, Spain; 4 Department of Automatic Control (ESAII), Biomedical Engineering Research Center (CREB), Universitat Politècnica de Catalunya (UPC), Barcelona, Spain; 5 Biomedical Research Networking Center in Bioengineering, Biomaterials and Nanomedicine (CIBER-BBN), Barcelona, Spain; 6 Departament de Farmacologia i Terapèutica, Universitat Autònoma de Barcelona, Barcelona, Spain; 7 Nuclear Medicine Department, Hospital de la Santa Creu i Sant Pau, Barcelona, Spain; 8 Centre d’Investigació de Medicaments, Sant Pau Institute of Biomedical Research (IIB-Sant Pau), Barcelona, Spain; 9 Servei de Farmacologia Clínica, Hospital de la Santa Creu i Sant Pau, Barcelona, Spain; 10 Centro de Investigación Biomédica en Red de Salud Mental, CIBERSAM, Spain; 11 Blanquerna School of Health Science, Universitat Ramon Llull, Barcelona, Spain; 12 Department of Neuropsychology and Psychopharmacology, Maastricht University, Maastricht,the Netherlands

**Keywords:** EEG, hallucinogens, Kappa opioid receptor, opioids, salvinorin-A, SPECT

## Abstract

**Background:**

The mechanisms through which kappa opioid receptor (KOR) agonists induce psychotomimetic effects are largely unknown, although the modulation of this receptor has attracted attention for its clinical use. In this work, we characterize the neuropharmacological effects of salvinorin-A, a highly selective KOR agonist.

**Methods:**

Changes in multimodal electroencephalography, single-photon emission computed tomography, and subjective effects following the acute administration of salvinorin-A are reported. The study included 2 sub-studies that employed a double-blind, crossover, randomized, placebo-controlled design.

**Results:**

The electroencephalography measures showed a marked increase in delta and gamma waves and a decrease in alpha waves while subjects were under the effect of salvinorin-A. Regarding single-photon emission computed tomography measures, significant decreases in regional cerebral blood flow were detected in multiple regions of the frontal, temporal, parietal, and occipital cortices. Significant regional cerebral blood flow increases were observed in some regions of the medial temporal lobe, including the amygdala, the hippocampal gyrus, and the cerebellum. The pattern of subjective effects induced by salvinorin-A was similar to those observed in relation to other psychotomimetic drugs but with an evidently dissociative nature. No dysphoric effects were reported.

**Conclusion:**

The salvinorin-A–mediated KOR agonism induced dramatic psychotomimetic effects along with a generalized decrease in cerebral blood flow and electric activity within the cerebral cortex.

**Significance Statement**

Kappa opioid receptors (KOR) remain largely unexplored for clinical use. The mechanisms underlying the effects of KOR agonists, including the associated psychotomimetic effects, remain elusive. In this study, we characterize the neuropharmacology of KOR for the first time, to our knowledge, using a combination of techniques, finding that the hallucinatory state induced by KOR full agonism is associated with a dramatic decrease in regional cerebral blood flow in the cortical areas of the brain.

## Introduction

Opioid receptors belong to the large family of G protein-coupled receptors, and they are expressed by central and peripheral neurons (Stein, 2016). They were named for their interactions with morphine, codeine, and other active principles found in opium, a medicinal product made with the latex of the plant *Papaver somniferum*. Despite opium having been used to treat pain and other symptoms for thousands of years, it was not until the 1970s that a group of membrane proteins with an affinity to morphine was discovered. Three subtypes were identified through binding studies and bioassays: µ (MOR), δ (DOR), and k (KOR) receptors (Brownstein, 1993).

Except in the case of endomorphins, most endogenous opioids are not selective for one of the opioid receptors. This is because opioid receptors share structural similarities, and they tend to form homomeric and heteromeric complexes with other opioid and non-opioid receptors. In this way, the effect of an opioid ligand is commonly the result of an interaction between different receptor complexes. In contrast, synthetic opioid peptides and alkaloids show selectivity for specific opioid receptors, and they have been used to characterize the pharmacological properties of opioid receptors (Feng et al., 2012; Valentino and Volkow, 2018). Although all three opioid receptors modulate pain and analgesia, MOR has been studied most extensively. MOR agonists not only have potent analgesic effects, but they also induce euphoria, respiratory depression, and physical and psychological dependence (Al-Hasani and Bruchas, 2011). To have potent analgesics without these side effects, several ligands with varying potencies, efficacies, and selectivity profiles for other opioid receptors have been designed. DOR agonists have anxiolytic, neuroprotective, and analgesic effects, but their activation may lead to convulsions, whereas KOR agonists have analgesic, antidepressant, and neuroprotective effects, but dysphoric and psychotomimetic effects are also common (Stein, 2016).

In recent years, interest in KOR agonists for the treatment of pain has increased considerably (Snyder et al., 2018) given the side effects associated with MOR agonists, such as euphoria and addiction. KOR ligands have also shown promising results for the treatment of depression and drug dependence (Butelman et al., 2012; Taylor and Manzella, 2016). However, as noted, the selective activation of KOR is associated with dysphoria and psychotomimetic effects (Clark and Abi-Dargham, 2019), although strategies have been developed in an effort to avoid these undesired effects (e.g., the use of peripherally restricted compounds). The mechanism through which selective KOR agonists induce dysphoria may involve the recruitment of β-arrestin and the activation of p38 mitogen-activated protein kinase. However, the mechanism through which psychotomimetic effects are induced remains unknown.

In human studies involving KOR agonists, hallucinations of monsters, disturbances of space and time, racing thoughts, and feelings of body distortion, among other effects, have been reported (Kumor et al., 1986; Pfeiffer et al., 1986). Recent clinical trials conducted by our group (Maqueda et al., 2015, 2016), in which salvinorin-A, a potent hallucinogenic non-nitrogenous diterpene that shows high selectivity for KOR (Roth et al., 2002) was used, have shown similar results. In these more recent studies, potent hallucinogenic effects were reported, consisting of changes in the perception of time, depersonalization, derealization, incapacity to interact with surroundings, and out-of-body experiences, among other effects. Although such experiences are all commonly classified as “hallucinogenic,” some study participants specified their uniqueness compared with effects induced by other hallucinogenic drugs (Maqueda et al., 2016). This may suggest a unique pattern of hallucinogenic effects elicited by the KOR agonism (Maqueda et al., 2015) in contrast with the effects of classical serotonergic psychedelics, which are mediated by 5-HT_2A_ receptors (Vollenweider et al., 1998; Kyzar et al., 2017; Holze et al., 2020). The study of salvinorin-A effects using neuroimaging techniques has been highly challenging given the fast onset and short duration of effects. An electroencephalography (EEG) study involving salvinorin-A reported a reduction in beta waves (Ranganathan et al., 2012). However, the participants in that study, who had previously used salvinorin-A, reported that the effect was equivalent to 20%–30% of the effect obtained in recreational contexts, suggesting the possibility that the researchers used a less efficient vaporization method, leading to lower absorption. Another salvinorin-A neuroimaging study used functional magnetic resonance imaging (fMRI) to analyze functional connectivity (FC) changes (Doss et al., 2020). It was found that the peak effects were associated with within-network decreases and between-network increases in static FC. The attenuation of the default mode network (DMN) FC was one of the most notable effects.

The aim of this study is to reveal functional changes in the brain associated with the hallucinogenic effects of the KOR agonism. Two independent, complementary studies were conducted with healthy volunteers. Sub-study 1 assessed spontaneous brain electrical oscillations using EEG while individuals were under the effect of salvinorin-A, whereas subjective measures and single-photon emission computed tomography (SPECT) were used in sub-study 2 to characterize both clinical effects and changes in regional cerebral blood flow (rCBF) following the acute administration of salvinorin-A.

## MATERIALS AND METHODS

### Ethics

This study was conducted in accordance with the Declarations of Helsinki and its updates concerning experimentation on humans, and it was approved by the Santa Creu i Sant Pau hospital’s ethics committee and the Spanish Ministry of Health. All participants gave their written informed consent prior to participation.

### Study Design, Sample, and Assessments

Volunteers with previous experience with hallucinogens (at least 10 previous experiences and no history of adverse effects) were recruited for both sub-studies. Participants underwent a clinical interview, physical examination, and laboratory testing, including hematology and urinalysis. Subjects with a history of psychiatric disorders, pregnancy, illness, or any substance dependence were excluded. Twenty-four people participated in sub-study 1 (EEG), while 20 people participated in sub-study 2 (SPECT). While in sub-study 1 the experimental feasibility was tested and only EEG measures were collected, the SPECT measures for sub-study 2 were complemented by the collection of subjective measures. One subject was removed from sub-study 1 as a consequence of the artifact rejection procedure, and 1 subject was removed from sub-study 2 due to technical problems that occurred while administering the substance, which resulted in the subject experiencing no psychoactive effects. Additional details on the study participants are provided in the supplementary Information file.

Both sub-studies were conducted following a double-blind, randomized, placebo-controlled design involving two experimental sessions that were one week apart. Volunteers were not allowed to take any medications or illicit drugs in the two weeks prior to beginning or during the sub-studies. The placebo or a fully psychoactive dose of 1 mg of vaporized pure (>99%) salvinorin-A, as based on a previous trial (Maqueda et al., 2015), was administered by inhalation. Based on previous studies (Maqueda et al., 2015, 2016), we expected the experience elicited by this dose to be remarkably intense. The psychoactive effects of salvinorin-A have a fast onset (approximately 1 minute) and short duration (10–15 minutes) and are characterized by vivid visions with eyes closed, changes in dimensionality and time perception, and a complete blockage of external stimuli (Maqueda et al., 2015, 2016).

### Subjective Measures

Psychological effects elicited by the administration of salvinorin-A were measured in sub-study 2 using the hallucinogen rating scale (HRS) questionnaire and various visual analogue scales (VAS).

The HRS has been widely used to assess the psychological effects of hallucinogenic drugs, including in previous studies conducted by our group (Riba et al., 2003; Maqueda et al., 2015, 2016). It includes 6 sub-scales: somesthesia, reflecting somatic effects; affect, showing sensitivity to emotional and affective responses; cognition, describing modifications in thought processes or content; perception, measuring visual, auditory, gustatory, and olfactory experiences; volition, indicating the volunteer’s capacity to willfully interact with his/her “self” and/or the environment; and intensity, which reflects the strength of the overall experience. A validated Spanish version of the questionnaire was used (Riba et al., 2001).

Self-administered VAS were used to retrospectively rate peak effects during the session. Volunteers retrospectively indicated the intensity of the drug effects using a chart with 100 horizontal lines placed 1 mm apart (from 0, no effects, to 100, extremely intense effects). There were 10 labeled lines for the VAS measures. “Global intensity” indicated the overall intensity of the experience. “Good effects” indicated any effect the volunteer perceived as positive. “I liked the experience” indicated the degree of enjoyment of the experience. “I would like to take the substance again” indicated willingness to take the substance again. “Bad effects” indicated any effect the volunteer perceived as negative. “Loss of contact with the body” indicated dissociation between mind and body. “Extracorporeal experiences” indicated the intensity of the sensation of being out-of-body. “Modification in time perception” indicated modifications of the perception of time. “Changes in dimensionality” indicated alterations in the perception of the dimensionality of the body. “Loss of contact with external reality” indicated separation from surroundings. “Visual phenomena” indicated visual modifications with eyes open or closed. “Auditory phenomena” indicated the intensity of possible sounds/noises/voices/music that the volunteer attributed to drug effects.

### EEG Data Collection and Processing

Vigilance-controlled EEG readings were recorded at baseline (for 3 minutes with eyes closed, 3 minutes before salvinorin-A vaporization), 0 minutes, +3 minutes, and +6 minutes after drug administration. EEG recordings were acquired via a BrainAmp amplifier (Brain Products GmbH, Gilching, Germany) using 19 electrodes according to the international 10/20 system (Fp1, Fp2, F7, F3, Fz, F4, T3, C3, Cz, C4, T4, T5, P3, Pz, P4, T6, O1, and O2) referenced to averaged mastoids. Vertical and horizontal electrooculograms were also recorded for ocular artifact minimization. Signals were recorded with a sample frequency of 100 Hz and analogically band-pass filtered between 0.1 and 45 Hz. EEG data was re-referenced to the common average. In previous studies with 10/20 systems, standardized LORETA (sLORETA) provided a solution with a localization error distance from the source of 1.90 ± 4.36 mm and a spread of 7.07 ± 0.37 mm (Song et al., 2015).

A 2-step procedure was applied to filter artifacts from EEG data. First, we applied an ocular contamination reduction process based on blind source separation (Romero et al., 2008). EEG signals were segmented into 5-second epochs. In the second stage, we applied an automatic rejection procedure focused on saturation, muscle, and movement artifacts (Anderer et al., 1992). A minimum of 6 artifact-free epochs was required to include a time-point EEG recording in the analysis. If the high-artifact time-point recording was the baseline, all of the volunteers would have been excluded from the analysis.

The sLORETA technique was applied to estimate 3D source distribution of the intracerebral current density function from the voltage values recorded by the scalp EEG electrodes. sLORETA estimates a particular solution of the non-unique EEG inverse solution restricted to 6239 cortical gray matter voxels with a spatial resolution of 0.125 cm^3^ according to a digitized head model from Montreal Neurological Institute (Pascual-Marqui, 2002). In the first step, the current density values were estimated based on the EEG cross-spectral matrix and then squared for each voxel in the classical frequency bands: delta (1.3–3.5 Hz), theta (3.5–7.5 Hz), alpha (7.5–13 Hz), beta (13–35 Hz), and gamma (35–40 Hz).

MATLAB and LORETA software were used to analyze the EEG signals. The MATLAB statistical toolbox was used for statistical analyses.

### Neuroimaging Acquisition and Processing

Brain 99mTc-D,L-hexamethylene-propyleneamine oxime SPECT scans were conducted prior to and following the administration of salvinorin-A using a 2-headed General Electric HELIX gamma camera equipped with fan-beam collimators. This technique is particularly appropriate given the extremely short nature of the psychotropic effects elicited by this drug, whereby the peak lasts for only 1 to 5 minutes following inhalation.

Subjects were trained to perform an uninterrupted inhalation of 30 seconds to absorb all of the vapor. Immediately following salvinorin-A inhalation (+25 seconds after the end of inhalation, i.e., at +55 seconds after the beginning of inhalation), the tracer was injected into each volunteer to precisely capture cerebral blood flow during the drug’s peak effect. Acquisition duration was roughly 50 minutes to achieve an appropriate signal-to-noise ratio. The initial tracer uptake and distribution, which reflects the rCBF at the time of injection, remain unchanged for at least 2 hours, independent of rCBF variations occurring after the fixation time. This property of quick uptake and prolonged stability is particularly indicated in this case, where the powerful effects of salvinorin-A are too short-lived to induce them and acquire brain scans using other techniques. Volunteers’ scans were acquired using a headrest and a 128 × 128 × 128 image matrix in 3-degree steps. Acquisition duration was roughly 50 minutes, and images were subsequently reconstructed using filtered back projection with the application of Metz filtering and attenuation-correction according to the Chang method, with a factor of 0.075.

Neuroimaging analyses were performed using the SPM8 software package (https://www.fil.ion.ucl.ac.uk/spm/). Briefly, the original SPECT images underwent the following preprocessing steps: DICOM to NIFTI conversion, spatial normalization to Montreal Neurological Institute standard anatomical space, and Gaussian smoothing using a kernel of 12 × 12 × 12 mm full-width at half maximum.

### Statistical Analyses

Post-salvinorin-A changes in subjective measures were assessed using paired *t* test analyses, for which a *P* < .001 after Bonferroni correction (.05/6 = .008 for the HRS questionnaire and .05/12 = .004 for VAS measures) was considered significant. Results are reported after Bonferroni correction.

Statistical EEG differences between drug and control groups were evaluated by paired-sample *t* tests computed for the baseline-corrected and log-transformed LORETA power values in each voxel and for each frequency band at different time points. To correct for multiple comparisons, a nonparametric permutation test based on the theory of randomization was applied (Nichols and Holmes, 2002). Voxel intensity nonparametric permutation test calculates a critical t-value by means of a random sample of all possible permutations in order to estimate the distribution of a maximum t-statistic.

Finally, to examine post-salvinorin-A rCBF changes, a SPECT voxel-wise paired *t* test was performed, using proportional scaling for global cerebral tracer uptake (Riba et al., 2006). Contrasts were selected to identify the set of voxels in which significant increases or decreases in tracer uptake were found following the administration of the drug compared with placebo. Only brain regions showing voxel-wise *P* < .001 and family-wise error correction for multiple comparisons (cluster-level *P* < .05 using random field theory as implemented in SPM) were considered significant.

## RESULTS

### Sub-study 1

A total of 24 participants (11 male, 13 female) were recruited, aged between 21 and 42 years (M = 33 years), with previous experience in the use of hallucinogenic drugs.

### EEG Data Acquisition and Analysis

**Figure 1.**
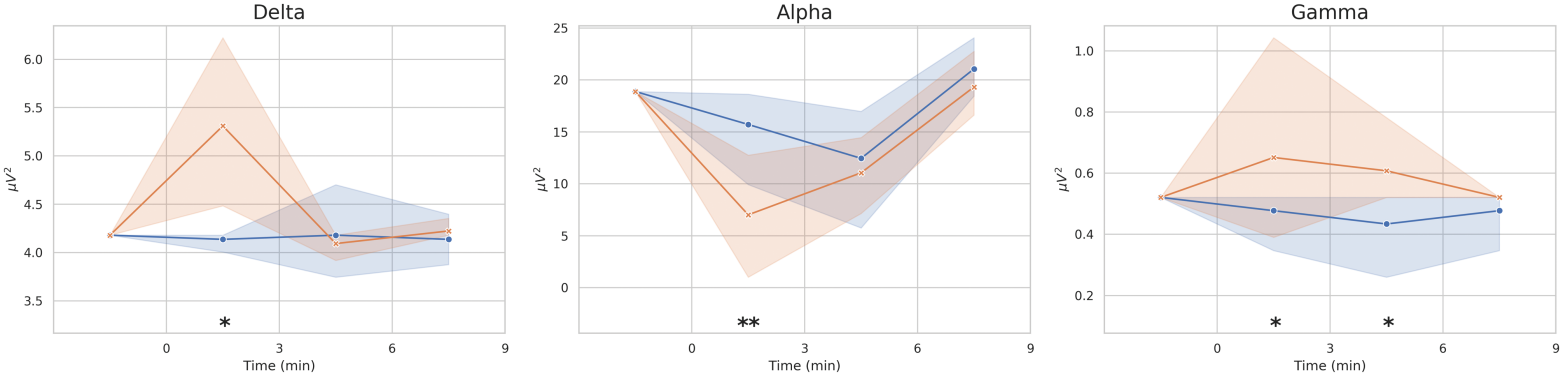
Averaged time courses (19 electrodes and 24 volunteers) of the absolute power (Y axis) in the delta, alpha, and gamma bands after placebo (blue) and salvinorin-A (SA; red) administrations. Shaded areas depict the SD of the mean in each time point. Significant changes are indicated by **P < .01 and *P < .05.

This study assessed the effect of a single dose of salvinorin-A (1 mg) using EEG measures. After the artifact rejection procedure, we established a minimum of 6 artifact-free epochs (30 seconds each) for each recording to obtain sufficient signal information to represent each time point. For this reason, 1 volunteer was excluded from the analysis. Figure 1 shows the time course for the main salvinorin-A effects measured by EEG. Alterations of absolute powers were highest in the first 3 minutes following the start of inhalation.

**Figure 2.**
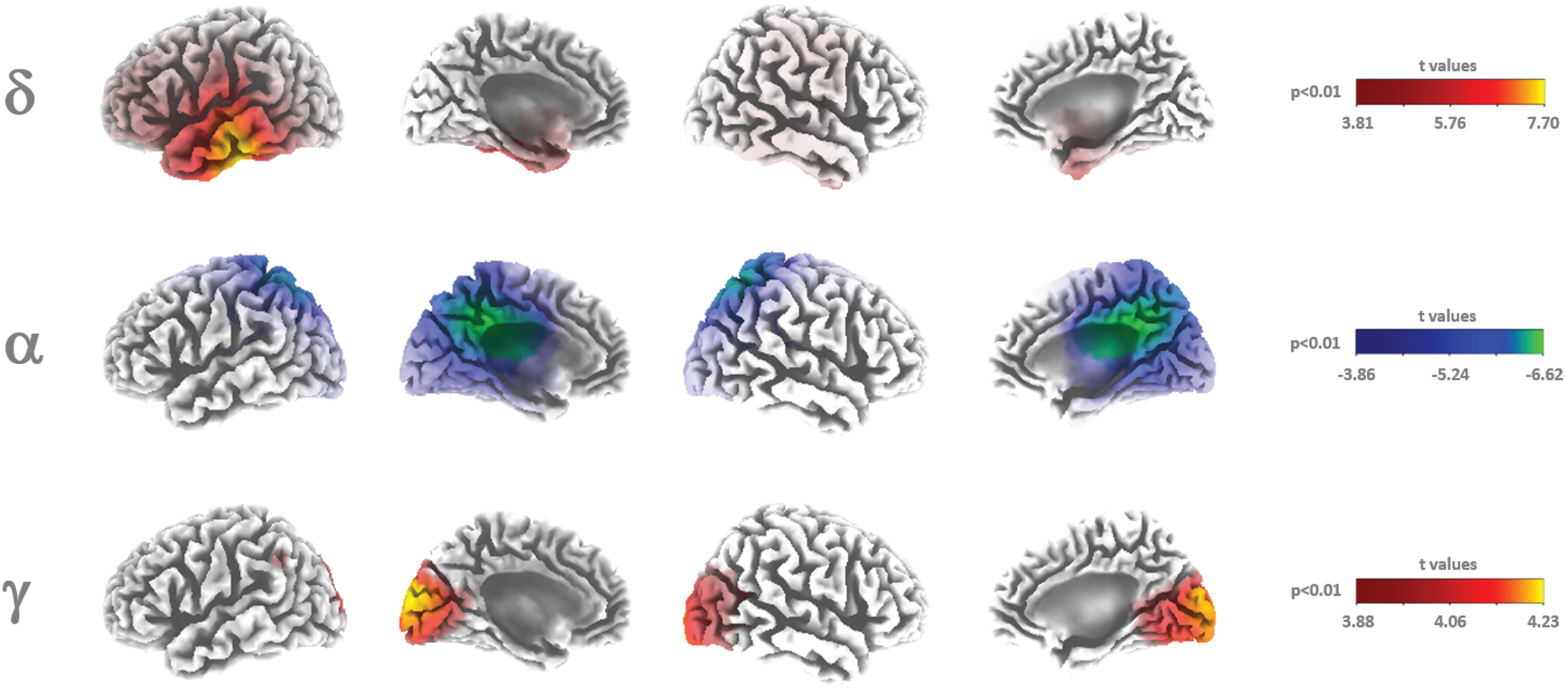
Three-dimensional LORETA statistical maps from the first 3 minutes after the acute inhalation of salvinorin-A (SA) compared with placebo for the delta (1.3–3.5 Hz), alpha (7.5–13 Hz), and gamma (35–40 Hz) frequency activities. Cold and hot colors indicate significant decreases and increases after voxel intensity nonparametric correction for multiple comparisons.

Neural sources associated with EEG frequency bands were estimated after placebo and salvinorin-A administrations. Figure 2 shows statistical LORETA images corresponding to suprathreshold regions found for delta, alpha, and gamma bands at the first 3 minutes after salvinorin-A administration as compared with the controls. Significant increases in delta and gamma bands were located at the temporal and occipital lobes, respectively. Decreases in the alpha band were restricted to the cingulate gyrus, precuneus, and superior parietal lobe.

### Sub-study 2

A sample of 20 participants (16 male, 4 female) was recruited, aged between 23 and 46 years (M = 35.1 years), with previous experience in the use of hallucinogenic drugs.

### Subjective Measures

#### HRS

**Figure 3.**
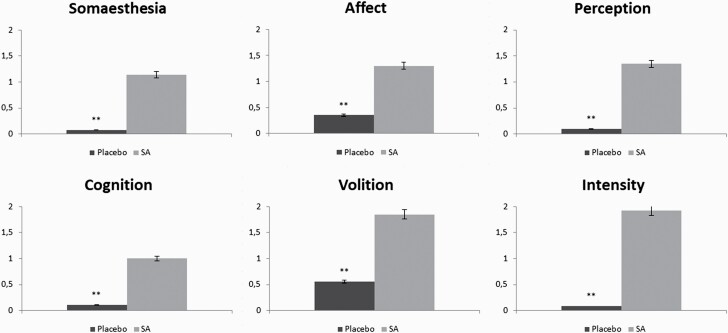
Comparison of scores in subscales of hallucinogen rating scale (HRS) questionnaire after the administration of salvinorin-A (SA) and placebo. ** *P* < .001.

**—**Mean scores obtained in the subscales of the HRS questionnaire for salvinorin-A and control conditions are shown in Figure 3. The scores for all subscales differed significantly between conditions, showing clear psychoactive effects induced by salvinorin-A. The subscales that showed higher post-salvinorin-A increases were intensity, volition, cognition, and perception.

#### VAS

**Figure 4.**
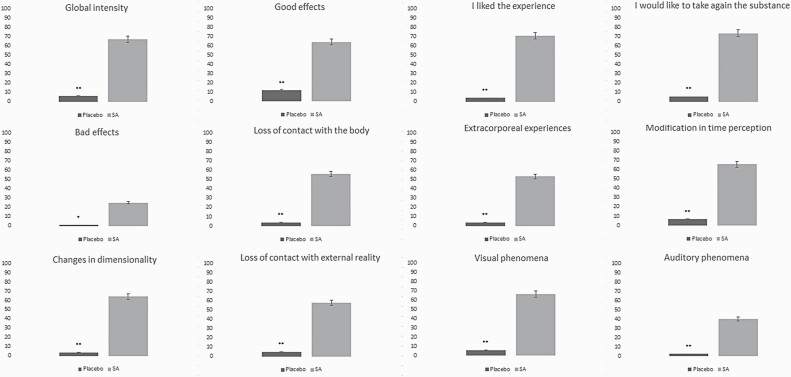
Comparison of scores in visual analogue scales (VAS) after the administration of salvinorin-A (SA) or placebo. *P = .001; **P < .001.

**—**Mean scores for all VAS are shown in Figure 4. Similar to the HRS, all items showed significantly higher scores following salvinorin-A administration than in the control condition. The highest scores were obtained for “I liked the experience,” “I would like to take the substance again,” “good effects,” and “changes in dimensionality.”

#### SPECT

**Figure 5.**
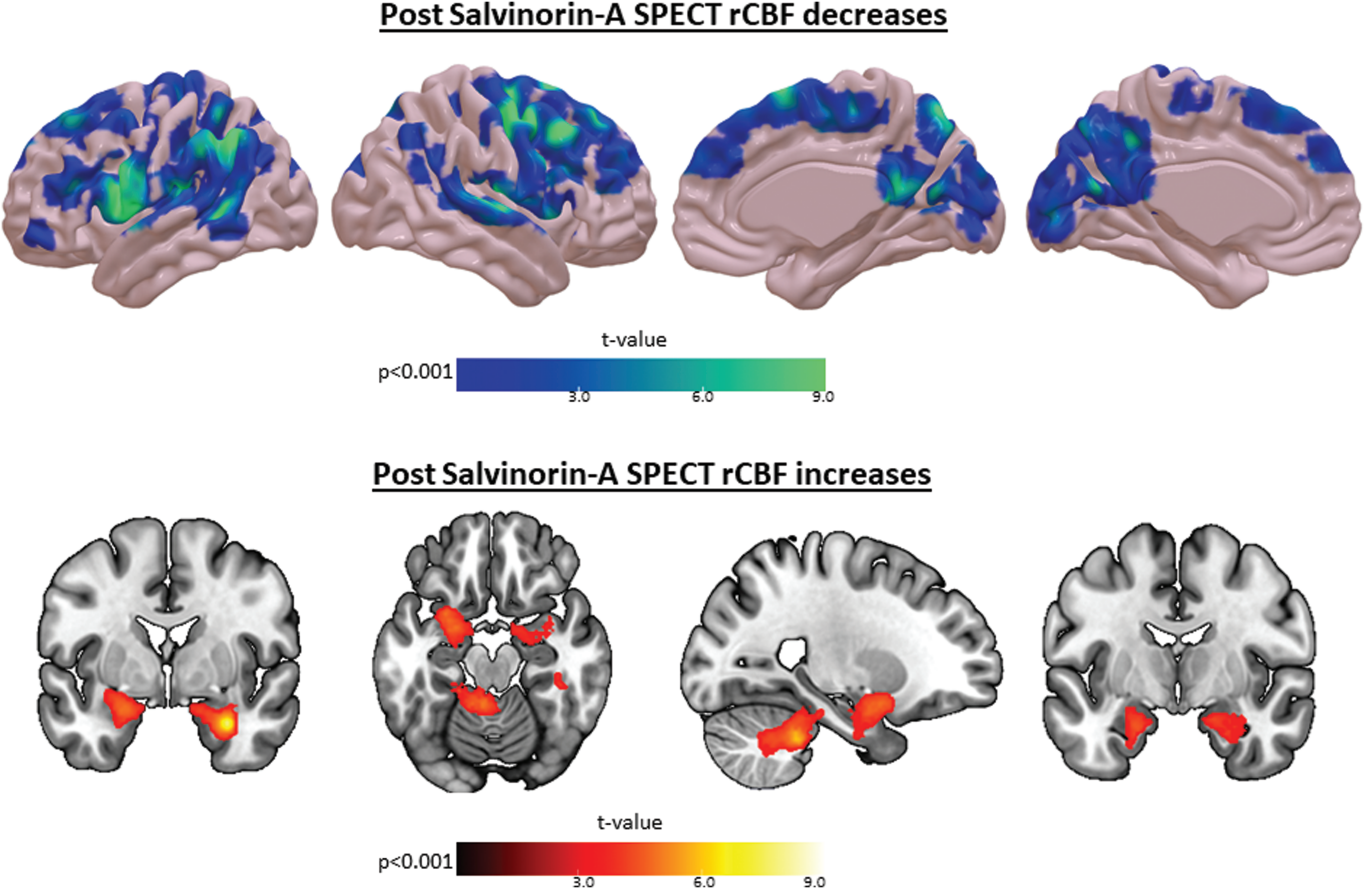
Decreases (above) and increases (below) in regional cerebral blood flow (rCBF) after the acute administration of salvinorin-A (SA). Only regions surviving voxel-wise *P < *.001 and cluster-level family-wise error correction for multiple comparisons (*P < *.05) are displayed.

Changes in rCBF produced by the acute administration of salvinorin-A are reported in Figure 5 (voxel-wise *P* < .001 and cluster-level family-wise error corrected *P* < .05). There was a dramatic and generalized decrease in rCBF in cortical areas, including mainly the frontal, temporal, and parietal lobes. Small decreases were also observed in regions of the occipital lobe, such as the calcarine sulcus. Increases in rCBF were located at the medial temporal lobe (especially in the amygdala, hippocampus, and parahippocampal gyrus) and the cerebellum.

## Discussion

The aim of this study was to characterize the neuropharmacological effects of salvinorin-A, a non-nitrogenous diterpene with high selectivity to KOR. As a consequence of the acute administration of salvinorin-A, drastic changes in subjective effects, as well as EEG and SPECT measures, were registered.

Regarding the subjective measures used in sub-study 2, the highest scores in HRS were obtained for the scales of intensity and volition. While the former concerns a very intense experience, the latter concerns a drastic interruption in contact with both the self (body sensations, awareness) and one’s surroundings. Among the scales with lower scores, we find affect, which suggests the low emotional content of the experience. While this pattern is different from that found in the case of psilocybin or ketamine (Gouzoulis-Mayfrank et al., 2005; Griffiths et al., 2011), some similarities exist with the pattern of effects obtained from using *N*,*N*-dimethyltryptamine (DMT) or ayahuasca (Riba et al., 2003). The battery of VAS showed the highest scores for “I liked the experience” and “I would like to take the substance again,” suggesting the mostly positive effects of salvinorin-A. This is in contrast with most KOR agonists, for which a pattern of dysphoric effects is generally reported (Kumor et al., 1986). However, it is possible that more dysphoric effects might be found if salvinorin-A were administered to naïve subjects (Butelman and Kreek, 2015). Other scales for which high scores were obtained include “visual effects,” “modifications in dimensionality,” and “loss of contact with external reality.” While alterations in visual stimuli are common with hallucinogenic drugs, modifications in dimensionality and the loss of contact with external reality are less common, except in the case of high doses of ketamine, which also induce out-of-body experiences (Morgan et al., 2009).

The EEG measures showed an increase in delta waves (mostly associated with human NREM sleep) along the temporal and parietal cortices and in limbic regions, such as the parahippocampal gyrus. This is in accordance with the subjective effects of salvinorin-A, which are commonly described as involving a dream-like state, usually with a loss of physical awareness (Addy, 2012; Maqueda et al., 2015). A marked decrease in alpha waves in the parietal and temporal lobes was also reported, possibly associated with the potent psychoactive effects of salvinorin-A and the subsequent loss of inhibitory control. Gamma waves showed enhanced activity mainly in the visual areas of the brain. These waves are associated with selective attention to sensory stimuli (Fries et al., 2001; Bichot et al., 2005). With eyes closed following the administration of hallucinogenic drugs, the activation of visual brain regions is similar to that observed when seeing a real image (de Araujo et al., 2012), so the enhanced activity of gamma waves could be attributed to being immersed in the intense visual imagery induced by salvinorin-A. Other EEG studies using serotonergic hallucinogens reported contradictory results, such as decreases in the power density of alpha and delta frequency bands (dos Santos et al., 2016). This suggests that different mechanisms of action underlie the hallucinogens’ psychotomimetic effects. Indeed, it is well known that salvinorin-A does not induce psychoactive effects through the 5-HT_2A_ receptors like classic psychedelic drugs do. Notably, a recent EEG study (Timmermann et al., 2019) is the only one to report similar findings. In this study, the authors reported reductions in alpha and beta waves and increases in delta and theta waves following the acute administration of DMT. This is consistent with the abovementioned similarities found between subjective measures for both drugs.

The administration of salvinorin-A was associated with a widespread cortical decrease in rCBF in the frontal, parietal, temporal, and occipital lobes. Post-salvinorin-A increases in rCBF were found in the cerebellum and in the medial regions of the temporal lobes of both hemispheres, especially in the amygdala, the hippocampus, and the parahippocampal gyrus, where there is a high density of KORs. This pattern differs again from that observed in other neuroimaging studies in which serotonergic hallucinogens were administered. For instance, a SPECT study involving mescaline reported a “hyperfrontal pattern,” the inverse situation (Hermle et al., 1992). Similarly, another SPECT study involving ayahuasca observed increases in CBF in the anterior insula, the inferior frontal gyrus, and the anterior cingulate, among other regions, with no region showing decreases in CBF (Riba et al., 2006). A positron emission tomography (PET) study offered similar results, showing increases in the cerebral metabolic rate of glucose in the frontomedial and frontolateral cortices, among other cortical regions, following the acute administration of psilocybin (Vollenweider et al., 1997). Conversely, Carhart-Harris et al. (2012) reported only decreases in CBF following acute administration of psilocybin. Those decreases were registered mainly in the thalamus and in the anterior cingulate and posterior cingulate cortices. However, it is challenging to compare these findings with ours given the distinct mechanism of action and subjective effects elicited by salvinorin-A. The main frontal regions showing decreases in CBF were the left prefrontal cortex, the superior frontal gyrus, and the premotor cortex, regions rich in KORs. It can be speculated that the decrease in CBF in these regions could explain the marked loss of contact with reality and body awareness reported by volunteers, but further research should clarify this finding. Regarding the parietal lobe, the precuneus and the supramarginal gyrus were the main regions to show decreases in CBF. It has been proposed that the precuneus might be involved in autobiographical memory and consciousness (Knauff et al., 2003; Cavanna, 2007; Freton et al., 2014). Furthermore, other neuroimaging studies have shown that the precuneus is among the first regions to be deactivated in other states of consciousness. Decreased activity in this region, during both slow-wave and REM sleep phases, has been reported in PET studies (Braun et al., 1997; Andersson et al., 1998), which may be related to the increased activity of delta waves. Another PET study found that the precuneus is among the first regions to show activity when individuals recover consciousness (Laureys et al., 1999). Additionally, it should be noted that the precuneus is a key node of the DMN. According to previous research (Carhart-Harris et al., 2012; Palhano-Fontes et al., 2015; Müller et al., 2018), decreased functional connectivity of the DMN is a common consequence of the acute administration of various hallucinogens, although there are some contradictory findings in this regard (Carhart-Harris et al., 2017; Müller et al., 2020).

Regarding the occipital lobe, the calcarine sulcus was the main region to show a relevant decrease in CBF. Interestingly, some previous studies involving hallucinogenic drugs found no effects in this region (Vollenweider et al., 1997; Riba et al., 2006; Carhart-Harris et al., 2016), whereas others found an increase in activity in this region (de Araujo et al., 2012; Carhart-Harris et al., 2016). This should raise questions about the different mechanisms through which hallucinatory experiences can be induced, as it has been proposed that psychedelic-induced hallucinations may be produced by the hyperactivation of visual areas of the brain; however, this is not applicable in the case of salvinorin-A. Moreover, activity in the calcarine sulcus has been considered essential to producing mental images (Ganis et al., 2004), so it is challenging to explain how most study participants could experience intense visual hallucinations while their visual cortices remained hypoactive. Again, further research using other neuroimaging techniques should shed light on this issue.

The increase in CBF registered in the medial temporal lobe, especially in the amygdala, suggests differential mechanisms with serotonergic hallucinogens. This appears to be the case as, following acute administration of both psilocybin and lysergic acid diethylamide, reduced activity and connectivity of the amygdala were observed (Kraehenmann et al., 2015; Mueller et al., 2017). The experience elicited by a high dose of salvinorin-A is much more abrupt and intense than that elicited by regular doses of serotonergic hallucinogens. We speculate that this may explain the increase of CBF in this region, as the amygdala is implicated in emotional salience. Moreover, dynamics between the corticofrontal regions and amygdala could also be involved, since attenuation of the activity of the amygdala has been associated with enhanced activation in some regions of the frontal cortex (Ochsner et al., 2002; Urry et al., 2006). The cerebellum also showed an increase in CBF. This could be interpreted in the context of well-known connections between the limbic system and the cerebellum (Blatt et al., 2013). Additionally, previously mentioned patterns that are similar to those found during sleep could also be related to this finding, as the cerebellum has been found to be increasingly active during sleep (Dang-vu et al., 2008). Nevertheless, we cannot be certain about these statements given the nature of the data obtained.

Among the limitations of this study, the small sample sizes used for both sub-studies should be mentioned. Moreover, the fact that participants had previous experience with hallucinogens could be related to the absence of dysphoric effects. Although the recruitment of naïve subjects for this kind of study is uncommon due to safety reasons, we should highlight this as a potential limitation of the whole body of neuropharmacological evidence regarding substances with psychedelic effects. Thus, the absence of dysphoric effects observed in this study should not be extended to samples with distinct profiles in terms of previous drug use. Additionally, we cannot rule out the possibility that the reported neuropharmacological findings are also inherent to people with previous experience with hallucinogens. Therefore, further research is needed to fully elucidate the pattern of dysphoric effects of salvinorin-A in the general population. Another limitation is the use of the HRS questionnaire, since its psychometric properties do not fit with the supposed number of scales, and thus we suggest interpreting the scales simply as an approximation of the actual pattern of subjective effects. Regarding the techniques used, SPECT is one of the few techniques that allows the observation of brain perfusion, being especially reliable and suitable in this case due to the fast-acting effects of salvinorin-A and the well-known procedure for “fixation” of the cerebral changes at the peak of the effects through the radiotracer. We would like to note that the combination of a technique with an excellent temporal resolution (EEG) and another technique measuring blood flow provides a highly unique framework for the characterization of the neuropharmacology of drugs with rapid onset and short duration of effects, such as salvinorin-A, DMT, 5-methoxy-*N*,*N*-dimethyltryptamine, and others that are currently being researched.

The discovery that salvinorin-A induces psychotomimetic effects through the signaling of KOR suggests that disorders of cognition and perception, such as schizophrenia, could be associated with alterations in this receptor system (Clark and Abi-Dargham, 2019). Beyond gaining an improved understanding of these pathological processes, the study of KOR signaling also offers an opportunity to elucidate the mechanisms of other superior functions, such as consciousness. In particular, it seems that KOR full agonists act as a kind of switch that completely interrupts perception and cognitive functions, inducing unique and bizarre hallucinations. The mechanism through which this phenomenon occurs, a drastic decrease in cortical rCBF, is reminiscent of the famous etching made by the Spanish painter Francisco Goya: “the sleep of reason produces monsters.”

## Supplementary Material

pyab063_suppl_Supplementary_File

## References

[CIT0001] Addy PH (2012) Acute and post-acute behavioral and psychological effects of salvinorin A in humans. Psychopharmacology 220:195–204. 21901316 10.1007/s00213-011-2470-6

[CIT0002] Al-Hasani R, Bruchas MR (2011) Molecular mechanisms of opioid receptor-dependent signaling and behavior. Anesthesiology 115:1363–1381. 22020140 10.1097/ALN.0b013e318238bba6 PMC3698859

[CIT0003] Anderer P, Semlitsch HV, Saletu B, Barbanoj MJ (1992) Artifact processing in topographic mapping of electroencephalographic activity in neuropsychopharmacology. Psychiatry Res 45:79–93. 1488471 10.1016/0925-4927(92)90002-l

[CIT0004] Andersson JL, Onoe H, Hetta J, Lidström K, Valind S, Lilja A, Sundin A, Fasth KJ, Westerberg G, Broman JE, Watanabe Y, Långström B (1998) Brain networks affected by synchronized sleep visualized by positron emission tomography. J Cereb Blood Flow Metab 18:701–715. 9663500 10.1097/00004647-199807000-00001

[CIT0005] Bichot NP, Rossi AF, Desimone R (2005) Parallel and serial neural mechanisms for visual search in macaque area V4. Science 308:529–534. 15845848 10.1126/science.1109676

[CIT0006] Blatt GJ, Oblak AL, Schmahmann JD (2013) Cerebellar connections with limbic circuits: anatomy and functional implications. In: Handbook of the Cerebellum and Cerebellar Disorders (Manto M, Schmahmann JD, Rossi F, Gruol DL, Koibuchi N, eds), pp. 479–496. Dordrecht: Springer. doi:10.1007/978-94-007-1333-8_22

[CIT0007] Braun AR, Balkin TJ, Wesenten NJ, Carson RE, Varga M, Baldwin P, Selbie S, Belenky G, Herscovitch P (1997) Regional cerebral blood flow throughout the sleep-wake cycle. An H2(15)O PET study. Brain 120 (Pt 7):1173–1197. 9236630 10.1093/brain/120.7.1173

[CIT0008] Brownstein MJ (1993) A brief history of opiates, opioid peptides, and opioid receptors. Proc Natl Acad Sci U S A 90:5391–5393. 8390660 10.1073/pnas.90.12.5391 PMC46725

[CIT0009] Butelman ER, Kreek MJ (2015) Salvinorin A, a kappa-opioid receptor agonist hallucinogen: pharmacology and potential template for novel pharmacotherapeutic agents in neuropsychiatric disorders. Front Pharmacol 6:190. 26441647 10.3389/fphar.2015.00190 PMC4561799

[CIT0010] Butelman ER, Yuferov V, Kreek MJ (2012) κ-opioid receptor/dynorphin system: genetic and pharmacotherapeutic implications for addiction. Trends Neurosci 35:587–596. 22709632 10.1016/j.tins.2012.05.005 PMC3685470

[CIT0011] Carhart-Harris RL, et al. (2016) Neural correlates of the LSD experience revealed by multimodal neuroimaging. Proc Natl Acad Sci U S A 113:4853–4858. 27071089 10.1073/pnas.1518377113 PMC4855588

[CIT0012] Carhart-Harris RL, Erritzoe D, Williams T, Stone JM, Reed LJ, Colasanti A, Tyacke RJ, Leech R, Malizia AL, Murphy K, Hobden P, Evans J, Feilding A, Wise RG, Nutt DJ (2012) Neural correlates of the psychedelic state as determined by fMRI studies with psilocybin. Proc Natl Acad Sci U S A 109:2138–2143. 22308440 10.1073/pnas.1119598109 PMC3277566

[CIT0013] Carhart-Harris RL, Roseman L, Bolstridge M, Demetriou L, Pannekoek JN, Wall MB, Tanner M, Kaelen M, McGonigle J, Murphy K, Leech R, Curran HV, Nutt DJ (2017) Psilocybin for treatment-resistant depression: fMRI-measured brain mechanisms. Sci Rep 7:13187. 29030624 10.1038/s41598-017-13282-7 PMC5640601

[CIT0014] Cavanna AE (2007) The precuneus and consciousness. CNS Spectr 12:545–552. 17603406 10.1017/s1092852900021295

[CIT0015] Clark SD, Abi-Dargham A (2019) The role of dynorphin and the kappa opioid receptor in the symptomatology of schizophrenia: a review of the evidence. Biol Psychiatry 86:502–511. 31376930 10.1016/j.biopsych.2019.05.012

[CIT0016] Dang-Vu TT, Schabus M, Desseilles M, Albouy G, Boly M, Darsaud A, Gais S, Rauchs G, Sterpenich V, Vandewalle G, Carrier J, Moonen G, Balteau E, Degueldre C, Luxen A, Phillips C, Maquet P (2008) Spontaneous neural activity during human slow wave sleep. Proc Natl Acad Sci U S A 105:15160–15165. 18815373 10.1073/pnas.0801819105 PMC2567508

[CIT0017] de Araujo DB, Ribeiro S, Cecchi GA, Carvalho FM, Sanchez TA, Pinto JP, de Martinis BS, Crippa JA, Hallak JE, Santos AC (2012) Seeing with the eyes shut: neural basis of enhanced imagery following Ayahuasca ingestion. Hum Brain Mapp 33:2550–2560. 21922603 10.1002/hbm.21381 PMC6870240

[CIT0018] dos Santos RG, Osório FL, Crippa JAS, Hallak JEC (2016) Classical hallucinogens and neuroimaging: a systematic review of human studies. Neurosci Biobehav Rev 71:715–728. 27810345 10.1016/j.neubiorev.2016.10.026

[CIT0019] Doss MK, May DG, Johnson MW, Clifton JM, Hedrick SL, Prisinzano TE, Griffiths RR, Barrett FS (2020) The acute effects of the atypical dissociative hallucinogen salvinorin A on functional connectivity in the human brain. Sci Rep 10:16392. 33009457 10.1038/s41598-020-73216-8 PMC7532139

[CIT0020] Feng Y, He X, Yang Y, Chao D, Lazarus LH, Xia Y (2012) Current research on opioid receptor function. Curr Drug Targets 13:230–246. 22204322 10.2174/138945012799201612 PMC3371376

[CIT0021] Freton M, Lemogne C, Bergouignan L, Delaveau P, Lehéricy S, Fossati P (2014) The eye of the self: precuneus volume and visual perspective during autobiographical memory retrieval. Brain Struct Funct 219:959–968. 23553546 10.1007/s00429-013-0546-2

[CIT0022] Fries P, Reynolds JH, Rorie AE, Desimone R (2001) Modulation of oscillatory neuronal synchronization by selective visual attention. Science 291:1560–1563. 11222864 10.1126/science.1055465

[CIT0023] Ganis G, Thompson WL, Kosslyn SM (2004) Brain areas underlying visual mental imagery and visual perception: an fMRI study. Brain Res Cogn Brain Res 20:226–241. 15183394 10.1016/j.cogbrainres.2004.02.012

[CIT0024] Gouzoulis-Mayfrank E, Heekeren K, Neukirch A, Stoll M, Stock C, Obradovic M, Kovar KA (2005) Psychological effects of (S)-ketamine and N,N-dimethyltryptamine (DMT): a double-blind, cross-over study in healthy volunteers. Pharmacopsychiatry 38:301–311. 16342002 10.1055/s-2005-916185

[CIT0025] Griffiths RR, Johnson MW, Richards WA, Richards BD, McCann U, Jesse R (2011) Psilocybin occasiones mystical-type experiences: immediate and persisting dose-related effects. Psychopharmacology 218:649–665. 21674151 10.1007/s00213-011-2358-5 PMC3308357

[CIT0026] Hermle L, Fünfgeld M, Oepen G, Botsch H, Borchardt D, Gouzoulis E, Fehrenbach RA, Spitzer M (1992) Mescaline-induced psychopathological, neuropsychological, and neurometabolic effects in normal subjects: experimental psychosis as a tool for psychiatric research. Biol Psychiatry 32:976–991. 1467389 10.1016/0006-3223(92)90059-9

[CIT0027] Holze F, Vizeli P, Ley L, Müller F, Dolder P, Stocker M, Duthaler U, Varghese N, Eckert A, Borgwardt S, Liechti ME (2020) Acute dose-dependent effects of lysergic acid diethylamide in a double-blind placebo-controlled study in healthy subjects. Neuropsychopharmacology 46:537–544. 33059356 10.1038/s41386-020-00883-6 PMC8027607

[CIT0028] Knauff M, Fangmeier T, Ruff CC, Johnson-Laird PN (2003) Reasoning, models, and images: behavioral measures and cortical activity. J Cogn Neurosci 15:559–573. 12803967 10.1162/089892903321662949

[CIT0029] Kraehenmann R, Preller KH, Scheidegger M, Pokorny T, Bosch OG, Seifritz E, Vollenweider FX (2015) Psilocybin-induced decrease in amygdala reactivity correlates with enhanced positive mood in healthy volunteers. Biol Psychiatry 78:572–581. 24882567 10.1016/j.biopsych.2014.04.010

[CIT0030] Kumor KM, Haertzen CA, Johnson RE, Kocher T, Jasinski D (1986) Human psychopharmacology of ketocyclazocine as compared with cyclazocine, morphine, and placebo. J Pharmacol Exp Ther 238:960–968. 3018228

[CIT0031] Kyzar EJ, Nichols CD, Gainetdinov RR, Nichols DE, Kalueff AV (2017) Psychedelic drugs in biomedicine. Trends Pharmacol Sci 38:992–1005. 28947075 10.1016/j.tips.2017.08.003

[CIT0032] Laureys S, Goldman S, Phillips C, Van Bogaert P, Aerts J, Luxen A, Franck G, Maquet P (1999) Impaired effective cortical connectivity in vegetative state: preliminary investigation using PET. Neuroimage 9:377–382. 10191166 10.1006/nimg.1998.0414

[CIT0033] Maqueda AE, Valle M, Addy PH, Antonijoan RM, Puntes M, Coimbra J, Ballester MR, Garrido M, González M, Claramunt J, Barker S, Johnson MW, Griffiths RR, Riba J (2015) Salvinorin-A induces intense dissociative effects, blocking external sensory perception and modulating interoception and sense of body ownership in humans. Int J Neuropsychopharmacol 18:pyv065. 26047623 10.1093/ijnp/pyv065 PMC4675976

[CIT0034] Maqueda AE, Valle M, Addy PH, Antonijoan RM, Puntes M, Coimbra J, Ballester MR, Garrido M, González M, Claramunt J, Barker S, Lomnicka I, Waguespack M, Johnson MW, Griffiths RR, Riba J (2016) Naltrexone but not ketanserin antagonizes the subjective, cardiovascular, and neuroendocrine effects of salvinorin-A in humans. Int J Neuropsychopharmacol 19:pyw016. 26874330 10.1093/ijnp/pyw016 PMC4966277

[CIT0035] Morgan CJ, Huddy V, Lipton M, Curran HV, Joyce EM (2009) Is persistent ketamine use a valid model of the cognitive and oculomotor deficits in schizophrenia? Biol Psychiatry 65:1099–1102. 19111280 10.1016/j.biopsych.2008.10.045 PMC2777248

[CIT0036] Mueller F, Lenz C, Dolder PC, Harder S, Schmid Y, Lang UE, Liechti ME, Borgwardt S (2017) Acute effects of LSD on amygdala activity during processing of fearful stimuli in healthy subjects. Transl Psychiatry 7:e1084. 28375205 10.1038/tp.2017.54 PMC5416695

[CIT0037] Müller F, Dolder PC, Schmidt A, Liechti ME, Borgwardt S (2018) Altered network hub connectivity after acute LSD administration. Neuroimage Clin 18:694–701. 29560311 10.1016/j.nicl.2018.03.005 PMC5857492

[CIT0038] Müller F, Holze F, Dolder P, Ley L, Vizeli P, Soltermann A, Liechti ME, Borgwardt S (2020) MDMA-induced changes in within-network connectivity contradict the specifics of these alterations for the effects of serotonergic hallucinogens. Neuropsychopharmacology 46:545–553. 33219313 10.1038/s41386-020-00906-2 PMC8027447

[CIT0039] Nichols TE, Holmes AP (2002) Nonparametric permutation tests for functional neuroimaging: a primer with examples. Hum Brain Mapp 15:1–25. 11747097 10.1002/hbm.1058 PMC6871862

[CIT0040] Ochsner KN, Bunge SA, Gross JJ, Gabrieli JD (2002) Rethinking feelings: an FMRI study of the cognitive regulation of emotion. J Cogn Neurosci 14:1215–1229. 12495527 10.1162/089892902760807212

[CIT0041] Palhano-Fontes F, Andrade KC, Tofoli LF, Santos AC, Crippa JA, Hallak JE, Ribeiro S, de Araujo DB (2015) The psychedelic state induced by ayahuasca modulates the activity and connectivity of the default mode network. PLoS One 10:e0118143. 25693169 10.1371/journal.pone.0118143 PMC4334486

[CIT0042] Pascual-Marqui RD (2002) Standardized low-resolution brain electromagnetic tomography (sLORETA): technical details. Methods Find Exp Clin Pharmacol 24 Suppl D:5–12. 12575463

[CIT0043] Pfeiffer A, Brantl V, Herz A, Emrich HM (1986) Psychotomimesis mediated by kappa opiate receptors. Science 233:774–776. 3016896 10.1126/science.3016896

[CIT0044] Ranganathan M, Schnakenberg A, Skosnik PD, Cohen BM, Pittman B, Sewell RA, D’Souza DC (2012) Dose-related behavioral, subjective, endocrine, and psychophysiological effects of the κ opioid agonist Salvinorin A in humans. Biol Psychiatry 72:871–879. 22817868 10.1016/j.biopsych.2012.06.012 PMC3638802

[CIT0045] Riba J, Rodríguez-Fornells A, Strassman RJ, Barbanoj MJ (2001) Psychometric assessment of the hallucinogen rating scale. Drug Alcohol Depend 62:215–223. 11295326 10.1016/s0376-8716(00)00175-7

[CIT0046] Riba J, Romero S, Grasa E, Mena E, Carrió I, Barbanoj MJ (2006) Increased frontal and paralimbic activation following ayahuasca, the pan-Amazonian inebriant. Psychopharmacology 186:93–98. 16575552 10.1007/s00213-006-0358-7

[CIT0047] Riba J, Valle M, Urbano G, Yritia M, Morte A, Barbanoj MJ (2003) Human pharmacology of ayahuasca: subjective and cardiovascular effects, monoamine metabolite excretion, and pharmacokinetics. J Pharmacol Exp Ther 306:73–83. 12660312 10.1124/jpet.103.049882

[CIT0048] Romero S, Mañanas MA, Barbanoj MJ (2008) A comparative study of automatic techniques for ocular artifact reduction in spontaneous EEG signals based on clinical target variables: a simulation case. Comput Biol Med 38:348–360. 18222418 10.1016/j.compbiomed.2007.12.001

[CIT0049] Roth BL, Baner K, Westkaemper R, Siebert D, Rice KC, Steinberg S, Ernsberger P, Rothman RB (2002) Salvinorin A: a potent naturally occurring nonnitrogenous kappa opioid selective agonist. Proc Natl Acad Sci U S A 99:11934–11939. 12192085 10.1073/pnas.182234399 PMC129372

[CIT0050] Snyder LM, et al. (2018) Kappa opioid receptor distribution and function in primary afferents. Neuron 99:1274–1288.e6. 30236284 10.1016/j.neuron.2018.08.044 PMC6300132

[CIT0051] Song J, Davey C, Poulsen C, Luu P, Turovets S, Anderson E, Li K, Tucker D (2015) EEG source localization: sensor density and head surface coverage. J Neurosci Methods 256:9–21. 26300183 10.1016/j.jneumeth.2015.08.015

[CIT0052] Stein C (2016) Opioid receptors. Annu Rev Med 67:433–451. 26332001 10.1146/annurev-med-062613-093100

[CIT0053] Taylor GT, Manzella F (2016) Kappa opioids, salvinorin A and major depressive disorder. Curr Neuropharmacol 14:165–176. 26903446 10.2174/1570159X13666150727220944 PMC4825947

[CIT0054] Timmermann C, Roseman L, Schartner M, Milliere R, Williams LTJ, Erritzoe D, Muthukumaraswamy S, Ashton M, Bendrioua A, Kaur O, Turton S, Nour MM, Day CM, Leech R, Nutt DJ, Carhart-Harris RL (2019) Neural correlates of the DMT experience assessed with multivariate EEG. Sci Rep 9:16324. 31745107 10.1038/s41598-019-51974-4 PMC6864083

[CIT0055] Urry HL, van Reekum CM, Johnstone T, Kalin NH, Thurow ME, Schaefer HS, Jackson CA, Frye CJ, Greischar LL, Alexander AL, Davidson RJ (2006) Amygdala and ventromedial prefrontal cortex are inversely coupled during regulation of negative affect and predict the diurnal pattern of cortisol secretion among older adults. J Neurosci 26:4415–4425. 16624961 10.1523/JNEUROSCI.3215-05.2006 PMC6673990

[CIT0056] Valentino RJ, Volkow ND (2018) Untangling the complexity of opioid receptor function. Neuropsychopharmacology 43:2514–2520. 30250308 10.1038/s41386-018-0225-3 PMC6224460

[CIT0057] Vollenweider FX, Leenders KL, Scharfetter C, Maguire P, Stadelmann O, Angst J (1997) Positron emission tomography and fluorodeoxyglucose studies of metabolic hyperfrontality and psychopathology in the psilocybin model of psychosis. Neuropsychopharmacology 16:357–372. 9109107 10.1016/S0893-133X(96)00246-1

[CIT0058] Vollenweider FX, Vollenweider-Scherpenhuyzen MF, Bäbler A, Vogel H, Hell D (1998) Psilocybin induces schizophrenia-like psychosis in humans via a serotonin-2 agonist action. Neuroreport 9:3897–3902. 9875725 10.1097/00001756-199812010-00024

