# Supplementary material for: The Kappa Opioid Receptor and the Sleep of Reason: Cortico-Subcortical Imbalance Following Salvinorin-A
Source: Int J Neuropsychopharmacol. 2021 Sep 19;25(1):54–63. doi: 10.1093/ijnp/pyab063 (PMC8756086; doi:10.1093/ijnp/pyab063)
Supplement: pyab063_suppl_Supplementary_File [file pyab063_suppl_supplementary_file.docx]

Supplementary information file

Sample

Sub-study 1

In this sub-study 24 volunteers with previous experience in the use of psychedelic drugs were recruited. Potential participants were interviewed by the principal investigator (JR) who recorded their previous experience with perception modifying drugs and explained the goals and methods of the study. The final participant sample had at least 10 previous experiences with psychedelics and no history of adverse effects from their use. Exclusion criteria included a current or past history of psychiatric disorders, alcohol or other substance dependence, evidence of significant illness, and pregnancy. Participants underwent a complete physical examination that included a medical history, laboratory tests, ECG, and urinalysis. Cannabis users were requested to abstain from cannabis use since enrollment and until the end of the study. This was verified by urinalysis.

Sub-study 2

In this sub-study 20 healthy volunteers with previous experience in the use of psychedelic drugs were recruited. The final participant sample had at least 10 previous experiences with psychedelics and no history of adverse effects from their use. Exclusion criteria were the same applied in sub-study 1. Participants underwent a complete physical examination that included a medical history, laboratory tests, ECG, and urinalysis. Cannabis users were requested to abstain from cannabis use since enrollment and until the end of the study. This was verified by urinalysis.

Description of subjective measures

The HRS has been widely used in the assessment of the psychological effects of various psychedelic drugs. This questionnaire was used in previous studies conducted by our group.^9,10,15,16^ It includes six sub-scales: somaesthesia, reflecting somatic effects; affect, showing sensitivity to emotional and affective responses; cognition, describing modifications in thought processes or content; perception, measuring visual, auditory, gustatory, and olfactory experiences; volition, indicating the volunteer’s capacity to willfully interact with his/her “self” and/or the environment; and intensity, which reflects the strength of the overall experience. A validated Spanish version of the questionnaire was used.^46^

Self-administered VAS were used to retrospectively rate peak effects during the session. They were 100 mm horizontal lines with which volunteers retrospectively indicated the intensity of the drug effects (from 0, no effects, to 100, extremely intense effects). There were ten labeled VAS lines. “Global intensity” indicated overall intensity of the experience. “Good effects” indicated any effect the volunteer assessed as positive. “I liked the experience” indicated the degree of enjoyment of the experience. “I would like to take the substance again” indicated the willingness to take the substance again. “Bad effects” indicated any effect the volunteer assessed as negative. “Loss of contact with the body” indicated dissociation between mind and body. “Extracorporeal experiences” indicated the intensity of the sensation of being out-of-body. “Modification in time perception” indicated modifications of the perception of time. “Changes in dimensionality” indicated alterations in the perception of the dimensionality of the body. “Loss of contact with external reality” indicated separation from surroundings. “Visual phenomena” indicated visual modifications with eyes open or closed. “Auditory phenomena” indicated the intensity of possible sounds/noises/voices/music that the volunteer attributed to drug effects.
